# Assessing the Relationship between Climate Variables and Hemorrhagic Fever with Renal Syndrome Transmission in Eastern China: A Multi-Cities Time Series Study

**DOI:** 10.1155/2023/5572334

**Published:** 2023-09-20

**Authors:** Yao Wang, Qing Duan, Bo Pang, Xueying Tian, Jing Ma, Wei Ma, Zengqiang Kou, Hongling Wen

**Affiliations:** ^1^Department of Epidemiology, School of Public Health, Cheeloo College of Medicine, Shandong University, Jinan 250012, China; ^2^Department of Microbiological Laboratory Technology, School of Public Health, Cheeloo College of Medicine, Shandong University, Jinan 250012, China; ^3^Infection Disease Control of Institute, Shandong Center for Disease Control and Prevention, Shandong Provincial Key Laboratory of Infectious Disease Prevention and Control, Jinan 250014, China

## Abstract

Hemorrhagic fever with renal syndrome (HFRS) is a climate-sensitive infectious disease. The effect of climatic drivers might predict and prevent HFRS, and understanding their relationship is urgently needed in the face of climate change. This study aimed to investigate the effect of meteorological factors on HFRS incidence. The random forest regression model, generalized additive model, and distributed lag nonlinear model (DLNM) were constructed to predict the importance, nonlinear trend and interaction effect, and exposure-lag effect of meteorological factors on HFRS incidence based on the data obtained in Shandong Province, China, 2013–2022. The most crucial meteorological factor was the weekly mean temperature. Interaction results showed that relative humidity affected HFRS incidence only under high or low-temperature conditions, and the effect of relative humidity with high and low pressure was the opposite. Using the median value as the reference, DLNM indicated that extremely low temperature had significant associations with HFRS at a lag of 3–5 weeks. Under extremely high temperatures, relative risks (RRs) became significantly high from a lag of 11 weeks, with the lowest value of 1.07 (95% CI: 1.00–1.13). RRs increased and then decreased with increasing mean temperature at lag 4 and 8 weeks, whereas at lag 12 and 16 weeks, the RRs gradually increased as the mean temperature climbed. This study demonstrates the complex relationship between meteorological factors and HFRS incidence. Our findings provide implications for the development of weather-based HFRS early warning systems.

## 1. Introduction

Hemorrhagic fever with renal syndrome (HFRS) is a rodent-borne infectious disease caused by hantaviruses, with five clinical phases in typical patients, including fever, hypotensive shock, oliguric, polyuric, and convalescent phases [1, 2]. HFRS has become a significant epidemic in Asia and Europe, with China being the most affected country, accounting for over 90% of all HFRS cases worldwide in the last few decades [3].

HFRS is considered a climatic-sensitive infectious disease, and it is becoming increasingly apparent that variations profoundly impact HFRS dynamics [4–8]. Prior studies had identified distinct climatic impacts that exhibited variations in geographical distribution [9–12]. HFRS was relatively more sensitive to weather variability in subtropical regions than in temperate regions [10]. A study of 254 cities in China indicated that meteorological factors' interaction and marginal effects on HFRS varied from region to region in China [9]. The highest risks of HFRS occurred at the 45^th^, 30^th^, and 20^th^ percentiles of mean temperature, rainfall, and relative humidity, respectively [13]. A study in northwestern Argentina showed that hantavirus transmission was positively associated with lagged precipitation and temperature using biannual and quarterly models. In contrast, the bimestrial model indicated a direct relationship with the rainfall; the temperature relationship was inverse in the second lag period [14]. The results in Huludao City found that the risk of HFRS infection reached its highest when both temperature and precipitation were high [7]. Tian et al. [15] found that HFRS outbreaks correlate with specific environmental conditionslow-summer temperatures and abundant summer precipitation. In South China, HFRS incidence was positively correlated with annual precipitation and absolute humidity [16]. Consequently, climatic variables may serve as indicators of human HFRS transmission risk. However, little is known about the effects of the sunshine hour, atmospheric pressure, and wind speed on HFRS incidence.

Shandong Province, one of the regions most severely affected by HFRS [17], lies in the transition between the humid subtropical and humid continental zones and has dry winter compared to other endemic areas with a fully humid climate [18]. Unique geographical and climatic environments may significantly affect the distribution and density of vectors of HFRS and HFRS incidence. Unfortunately, relevant studies and knowledge still need to be made available. In this study, we used the random forest regression model [19], generalized additive model (GAM) [20], and distributed lag nonlinear model (DLNM) [21] to explore the importance, nonlinear trend and interaction effect, and exposure-lag effect of mean temperature, relative humidity, sunshine hour, precipitation, atmospheric pressure, and wind speed on HFRS incidence in Shandong Province. Our study may further inform scenario plans for climate-driven transmission control and upgrade local health care services and policy action.

## 2. Materials and Methods

### 2.1. Study Area

**Figure 1:**
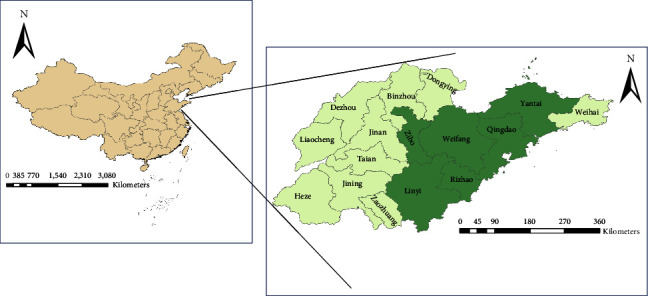
Location of Shandong Province in China. The green region is the study area.

Shandong Province is on the eastern edge of the North China Plain between longitudes 114°47.5′ *E*–122°42.3′ *E* and latitudes 34°22.9′ *N*–38°24.01′ *N* (Figure 1). Shandong has a temperate climate, lying in the transition between the humid continental and humid subtropical zones with four distinct seasons [18]. Shandong is divided into 16 prefecture-level divisions. This study included six cities (Qingdao, Zibo, Yantai, Weifang, Rizhao, and Linyi) with high-HFRS incidence (Figure 1).

### 2.2. Data Collection

By law, HFRS cases must be reported to China Information System for Diseases Control and Prevention (CISDCP) within 24 hr after diagnosis. The daily reported cases of HFRS from January 1, 2013 to December 31, 2022, were obtained from CISDCP. Meteorological data over the same period, including mean temperature (degrees Celsius, °C), mean windspeed (meters/second, m/s), atmospheric pressure (hPa), relative humidity (percentage, %), precipitation (millimeters, mm), and sunshine duration (hours, hr) were calculated from the corresponding daily data obtained from China Meteorological Data Sharing Service System (http://data.cma.cn/).

### 2.3. Data Analysis

A descriptive analysis was first performed to describe the temporal trend of HFRS cases and meteorological factors during the study period. A random forest regression model was performed to detect the importance of meteorological factors on HFRS incidence. Predictions were given by the averaged values or majority votes of each tree's prediction. “Out-of-bag” (OOB) samples were used for an unbiased estimate of the prediction error. The importance scores for the variables were computed by averaging the difference in OOB error before and after the permutation. The increase in the mean of squared residuals (%IncMSE) was used to measure the importance of the variable. Spearman correlation analysis was performed to evaluate the relationships among meteorological factors. To avoid collinearity, highly correlated variables (*r* > 0.6) were not included in the subsequent models simultaneously.

In this study, GAM was used to explore meteorological factors' nonlinear and interaction effects on the HFRS epidemic. We assumed that the weekly HFRS cases approximately followed a quasi-Poisson distribution and constructed a model between the logarithm of expected HFRS cases and meteorological variables through a GAM with a family of Gaussian distribution. Generalized cross-validation criteria estimated the optimal degrees of freedom (df) for the spline function. We calibrated the model using temporal trends as a confounder. The model was defined as follows:

(1) $$\begin{array}{c} \log Y_{t}=\alpha +s\;\left({\text{MF}}_{t},\text{df}\right)+s\left(\text{Week},\text{df}\right), \end{array}$$

where log (*Y_t_*) is the log transformed of the number of HFRS cases per week. *α* represents the intercept. MF_*t*_ is the meteorological factor of the week. *s* indicates penalized spline function. The week represents the number of weeks appearing in the HFRS cases.

Previous studies have demonstrated that the influence of meteorological variables on infectious diseases has a lag effect [7, 22, 23]. However, GAM only takes into account the effects within a particular period. DLNM is based on a combination of GAM and a distributed lag linear model, which adds a lag dimension to the exposure-response relation through the cross-basis function and simultaneously evaluates the lag effect and nonlinear effects. Relative risks (RRs) were calculated to assess the impact of meteorological factors with different lag weeks on HFRS incidence. The model can be written as follows:

(2) $$\begin{array}{c} \log\;E\left(Y_{t}\right)=\alpha +\text{cb}\left(M,{\text{df}}_{1}\right)+\text{ns}\left({\text{Cov}}_{t},{\text{df}}_{2}\right)+\text{ns}\left(\text{Week},{\text{df}}_{3}\right), \end{array}$$

where *E*(*Y_t_*) denotes the number of weekly reported HFRS cases at week t; *α* is the intercept; cb means the cross-basis function of the meteorological variable. In this study, *M* represents mean temperature; week is the ordinal variable of the week in the year to control the long temporal trend; ns represents the nature spline function; Cov is the covariate variable to control the impact of other meteorological factors on week *t*. This study set the maximum lag period to 16 weeks [7, 8].

For the DLNM analysis, we also performed several sensitivity analyses. We first included windspeed in the model. Then we changed the df for relative humidity (4), mean temperature (4), and week (7 and 9) to assess the robustness of DLNM.

All analyses were performed using the “caret,” “randomForest,” “rfPermute,” “corrplot,” “dlnm,” and “mgcv” packages in R software (version 4.1.0). All two-sided statistical tests' confidence interval (CI) was 95%, and *P* < 0.05 was considered statistically significant.

## 3. Results

### 3.1. Summary of Meteorological Factors and HFRS Cases

A total of 7,573 HFRS cases were reported during the study period. The weekly average values of HFRS cases, mean temperature, atmospheric pressure, sunshine duration, wind speed, relative humidity, and precipitation were 12.8 cases, 13.8°C, 995.6 hpa, 4.4 hr, 2.9 m/s, 63.6%, and 1.8 mm, respectively (Table S1). The temporal trends of HFRS cases and meteorological factors are shown in Figure S1, indicating a potential association between meteorological factors and HFRS incidence and seasonal HFRS case distribution pattern.

### 3.2. Relationship between Meteorological Factors and HFRS Incidence

**Figure 2:**
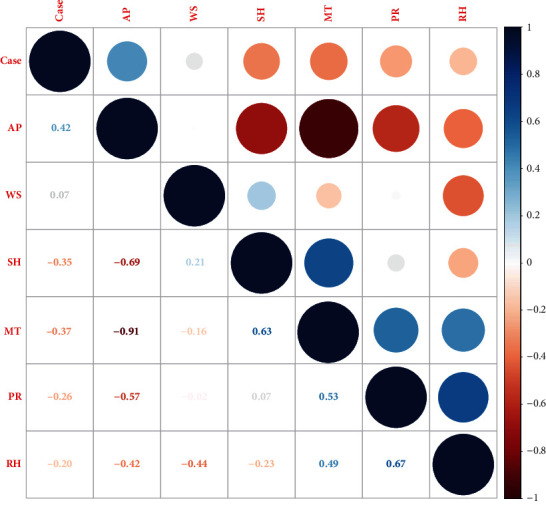
Spearman correlation coefficients between meteorological factors and weekly cases of HFRS. AP, weekly mean atmospheric pressure; WS, weekly mean wind speed; SH, weekly mean sunshine duration; MT, weekly mean temperature; PR, weekly mean precipitation; RH, weekly mean relative humidity.

Spearman correlation analysis indicated that atmospheric pressure (*r* = 0.42) was positively correlated with the number of HFRS cases (Figure 2); in contrast, mean temperature (*r* = −0.37), relative humidity (*r* = −0.20), rainfall (*r* = −0.26), and sunshine duration (*r* = −0.35) was negatively correlated with the incidence of HFRS (Figure 2).

**Figure 3:**
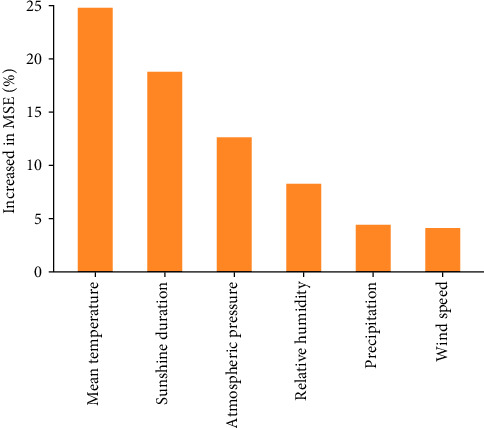
Importance of the meteorological factors on HFRS incidence.

Random forest regression analysis suggested that the essential meteorological factor for HFRS was mean temperature, with a %IncMSE greater than 20%, followed by sunshine duration (18.8%), atmospheric pressure (12.6%), and relative humidity (8.3%) (Figure 3). The top four variables were further analyzed in subsequent models.

**Figure 4:**
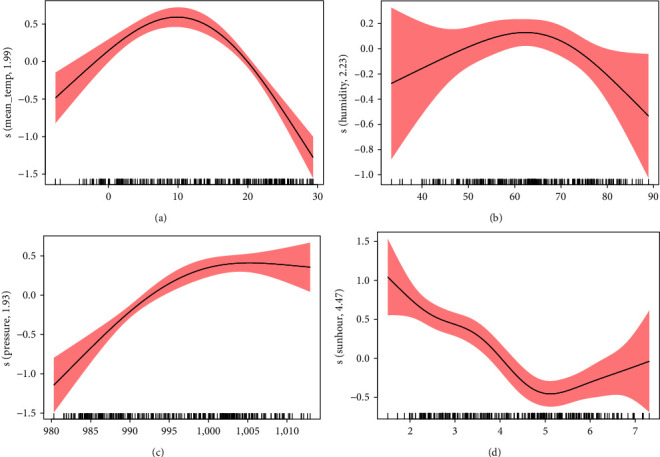
Exposure-response curves for the effects of meteorological factors on weekly HFRS cases in the univariate GAM. The *x*-axis is the meteorological parameters. The *y*-axis indicates the contribution of smoother to the fitted values. (a) Mean temperature, (b) relative humidity, (c) atmospheric pressure, and (d) sunshine duration.

GAM was constructed to perform univariate analyses for different meteorological factors (Figure 4), consistent with the Spearman correlation. The risk of HFRS descended with a mean temperature over 10°C. At atmospheric pressure below 1,005 hPa, the risk of HFRS incidence rose with the increased pressure. Conversely, the risk of HFRS incidence decreased with the increase in sunshine duration when its value was less than 5 hr. When relative humidity was above 65%, higher relative humidity restricted the occurrence of HFRS. The results of multivariable GAM were parallel to univariate GAM (Figure S2).

**Figure 5:**
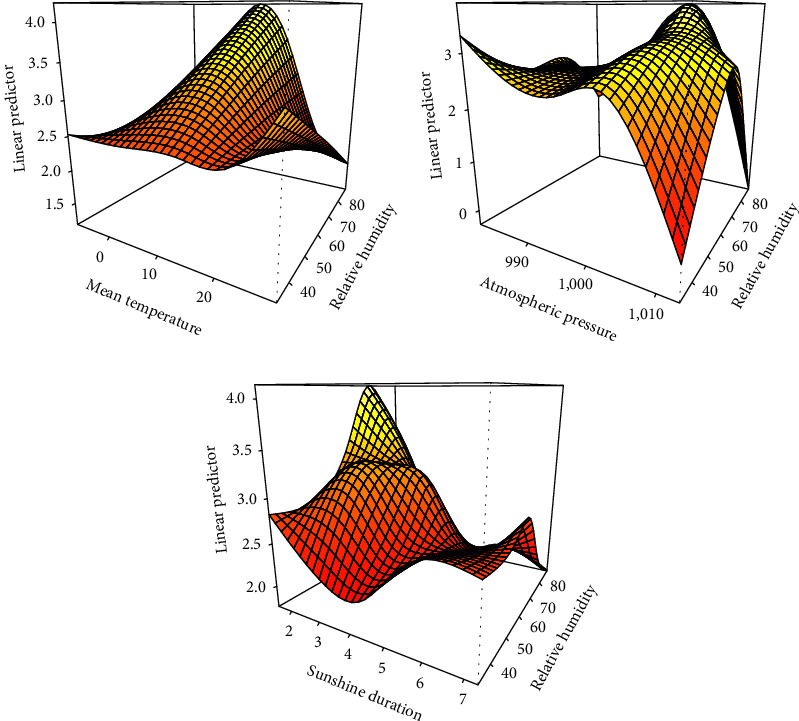
The effect interactions of the association between meteorological factors.

We also explored the interaction effect of mean temperature, sunshine duration, atmospheric pressure, and relative humidity on HFRS incidence. A significant interaction was observed between mean temperature, atmospheric pressure, sunshine duration, and relative humidity (Table S2, Figure 5). High temperatures with low-relative humidity increased the risk of HFRS incidence. Lower relative humidity contributed to HFRS incidence at low-atmospheric pressure, whereas higher relative humidity had the opposite effect. When relative humidity was around 60%, HFRS tended to occur at lower sunshine duration.

**Figure 6:**
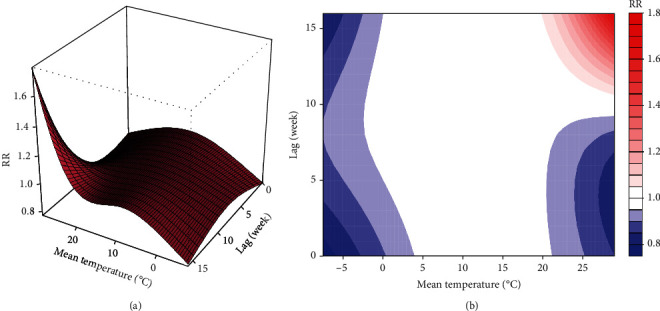
The 3D graphs and contour plots of mean temperature: (a) 3D plot and (b) contour plot.

**Figure 7:**
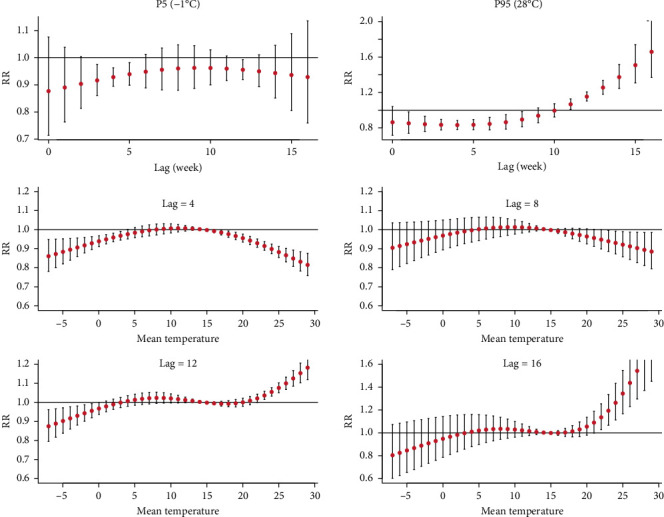
The exposure-lag effects of mean temperature on HFRS incidence using DLNM.

We further evaluated the lag effects of mean temperature using DLNM. We set the median temperature value as the reference (14.6°C), and then RRs with 95% confidence intervals of HFRS incidence among lag weeks were calculated with the 95^th^ (28°C) and 5^th^ (−1°C) percentile of mean temperature. We did the 3D and contour plots of mean temperature to comprehensively summarize the exposure-lag-response association (Figure 6). Generally, the effect of higher temperature with more extended lag periods increased significantly. Under the extremely low temperature (−1°C), the RRs for lag 3–5 weeks were significantly low, with the lowest effect estimate occurring on the lag 3^rd^ week (RR = 0.92, 95%CI: 0.86–0.98) (Figure 7). Furthermore, under the extremely high temperature (28°C), the RRs became significantly higher from lag 11^th^ week with the lowest value of 1.07 (95% CI: 1.00–1.13) (Figure 7).

Figure 7 shows the lag-specific association between mean temperature and HFRS incidence. The slice plots indicated that the RRs first increased and then decreased with increasing mean temperature at lag 4^th^ and 8^th^ week. At lag 12^th^ and 16^th^ weeks, the RRs gradually rose with the mean temperature increase.

The nonlinear trends and exposure-lag effects of sensitivity analyses were consistent with our model, indicating that our model was robust and reliable (Figures S3–S7).

## 4. Discussion

This study investigated the relationship between meteorological factors and HFRS incidence in Shandong Province using a random forest regression model, GAM, and DLNM. The results showed that meteorological factors contributed to HFRS incidence with nonlinear and complex patterns, with the mean temperature being the most important. Meteorological factors had significant interaction and lag effects on HFRS incidence.

Many factors influence HFRS, such as climate, social–economic, ecology, and rodent virus carrier rate [23, 24]. Climate change has been recognized as an essential driving factor influencing vector-borne diseases. It directly or indirectly affects the pathogen, vector, host, and susceptible population of infectious diseases and subsequently changes their mode, frequency, and intensity [25]. The propagation and expansion of pathogens in vectors are susceptible to meteorological factors [5].

Temperature is the most widely studied meteorological factor for climate-sensitive infectious diseases. It is one of the essential meteorological factors affecting HFRS incidence, with a complicated and nonlinear relationship in previous studies [5, 7, 8, 26]. Our results found that the exposure–response curve for temperature-HFRS incidence was approximately reversed U-shaped, in agreement with an earlier study [27]. The risk of HFRS incidence was high when the temperature was around 10°C. This temperature corresponds to the spring and autumn seasons in Shandong Province, coinciding with the HFRS incidence peak [17, 28]. HFRS is most common from late autumn until the following spring, with two peaks in incidence [17, 28]. The onset of the HFRS epidemic is determined by the type of pathogenic hantavirus and is associated with increased human outdoor activity in the spring and fall [29]. Both the Hantaan virus and Seoul virus strains have spread across Shandong, and the two virus strains can exist in the same area or different areas separately, leading to spring outbreaks or autumn outbreaks [30]. Temperature can affect disease dynamics through its effect on the dynamics of the rodent reservoir population and pathogen survival in the external environment and subsequently on human–animal contact patterns [11]. A previous study showed that rat density was negatively correlated with temperature [31]. Tian et al. [15] investigation revealed that temperature affected HFRS incidence in several ways, including rodent species' survival rate and density, outdoor human engagement, and hence the transmission of virus strains [11]. We also estimated the weekly lag effect of mean temperature on the occurrence of HFRS and found that the lag effects varied widely depending on temperature values and lag weeks. The different lag weeks reflected that the delayed effect might be related to the spread of the infection being affected by various factors, including the proliferation of the virus in the external environment, the tendency of people to go out, seasonal variability in the rodent populations [7]. Sun et al. [7] found a significant association between high temperatures and HFRS at 15 and 16 weeks lags. Joshi et al. [32] investigated the influence of climatic factors on the development of HFRS during the peak season and found that temperatures at a lag of 11 weeks had the largest RR. Xu et al. [27] indicated that the highest risk of HFRS incidence was found at a daily mean temperature of 10°C (lag 0) compared with the median temperature.

Our study found that high-relative humidity was unfavorable for HFRS incidence, in agreement with a previous study conducted in Weifang [27] but contrary to Guangdong's result [33]. This discrepancy may be attributed to different climatic and geographical environments. Shandong Province transitions between the humid subtropical and humid continental zones with dry, long, and cold winters [18]. Notably, higher humidity may enhance the infectivity and vigor of Hantavirus in the external environment, and wet or subhumid areas can promote HFRS transmission [34]. The above distinction motivates us to prioritize local areas rather than larger spatial scales. Compared to temperature and relative humidity, the effects of sunshine duration and atmospheric pressure have yet to be investigated. We found that higher atmospheric pressure contributed to HFRS incidence, while HFRS incidence was negatively associated with sunshine duration. Considering that the time series of mean temperature and sunshine duration are in phase, the effect of sunshine duration may be an indirect function of mean temperature. Sunshine duration influences HFRS transmission by affecting crop yield, rodent reproduction, and vector density [26]. In short, more studies are needed to investigate the mechanisms behind these relationships.

The interaction analysis results in our study demonstrated complex and integrated effects of meteorological factors on HFRS incidence. Relative humidity played different roles when mean temperature, atmospheric pressure, and sunshine duration were in different ranges. Sun et al. [7] found that the risk of HFRS infection was highest when both temperature and precipitation were high. In Taizhou, the risk of HFRS increased with the decreasing average temperature and the increasing precipitation [8]. A geographic distribution analysis indicated that the highest incidence of HFRS was reported in China's semihumid climate with a mountainous geographical structure [35].

This study has several strengths. First, among several meteorological factors examined, we pinpointed the one exerting the most substantial influence on the occurrence of HFRS. Second, our study included sunshine duration and atmospheric pressure, which were rarely analyzed in previous studies. Third, our time series of HFRS infections was based on the date of disease onset, minimizing errors due to notification delays.

Some limitations of our study should be noted. First, the collection of HFRS cases relied on passive surveillance, which may lead to underreporting due to the lack of medical facilities and limited detection capability. Fortunately, this situation has improved remarkably in recent years. Second, with China's rapid urbanization, economic, and demographic factors may also play a role in HFRS incidence. Third, the results from this study may not apply to other regions with different climates and geographical conditions.

## 5. Conclusions

The relationship between meteorological factors and HFRS was nonlinear, with significant lag and interaction effects. Our study provides information to understand better, the impact of meteorological factors variation on HFRS incidence and may inform resource allocation plans for disease control in the climate change scenarios.

## Data Availability

HFRS data underlying the results presented in the study cannot be shared publicly because of the limitation of data availability in the data management rule of the Shandong Center for Disease Control and Prevention. The corresponding author can provide additional information on the process upon request.

## Supplementary Materials

Supplementary Materials Table S1: descriptive statistics of weekly HFRS cases and meteorological factors. Table S2: model test of the interaction analysis. Figure S1: time series plot of HFRS cases and meteorological factors. Figure S2: exposure-response curves for the effects of meteorological factors on weekly HFRS cases in the multiple-variables model using GAM. The *x*-axis is the meteorological parameters. The *y*-axis indicates the contribution of the smoother to the fitted values. Figure S3: sensitivity analysis for DLNM by setting the degree of freedom of mean temperature to 4. Figure S4: sensitivity analysis for DLNM by setting the degree of freedom of relative humidity to 4. Figure S5: sensitivity analysis for DLNM by setting the degree of freedom of the week to 7. Figure S6: sensitivity analysis for DLNM by setting the ing degree of freedom of week to 9. Figure S7: sensitivity analysis for DLNM by including mean windspeed.

## References

[1] Schmaljohn C., Hjelle B. (1997). Hantaviruses: a global disease problem. *Emerging Infectious Diseases*.

[2] Kim H. K., Chung J.-H., Kim D.-M., Yun N.-R., Kim C.-M., Jalal S. (2019). Hemorrhagic fever with renal syndrome as a cause of acute diarrhea. *The American Journal of Tropical Medicine and Hygiene*.

[3] Zou L.-X., Chen M.-J., Sun L. (2016). Haemorrhagic fever with renal syndrome: literature review and distribution analysis in China. *International Journal of Infectious Diseases*.

[4] Roda Gracia J., Schumann B., Seidler A. (2015). Climate variability and the occurrence of human puumala hantavirus infections in Europe: a systematic review. *Zoonoses and Public Health*.

[5] Liu Q.-Y. (2021). Impact of climate change on vector-borne diseases and related response strategies in China: major research findings and recommendations for future research. *Chinese Journal of Vector Biology and Control*.

[6] Guan P., Huang D., He M., Shen T., Guo J., Zhou B. (2009). Investigating the effects of climatic variables and reservoir on the incidence of hemorrhagic fever with renal syndrome in Huludao City, China: a 17-year data analysis based on structure equation model. *BMC Infectious Diseases*.

[7] Sun W., Liu X., Li W., Mao Z., Sun J., Lu L. (2021). Effects and interaction of meteorological factors on hemorrhagic fever with renal syndrome incidence in Huludao City, northeastern China, 2007–2018. *PLOS Neglected Tropical Diseases*.

[8] Zhang R., Zhang N., Sun W. (2022). Analysis of the effect of meteorological factors on hemorrhagic fever with renal syndrome in Taizhou City, China, 2008–2020. *BMC Public Health*.

[9] Cao L., Huo X., Xiang J. (2020). Interactions and marginal effects of meteorological factors on haemorrhagic fever with renal syndrome in different climate zones: evidence from 254 cities of China. *Science of the Total Environment*.

[10] Xiang J., Hansen A., Liu Q. (2018). Impact of meteorological factors on hemorrhagic fever with renal syndrome in 19 cities in China, 2005–2014. *Science of the Total Environment*.

[11] Tian H., Stenseth N. C. (2019). The ecological dynamics of hantavirus diseases: from environmental variability to disease prevention largely based on data from China. *PLOS Neglected Tropical Diseases*.

[12] Carver S., Mills J. N., Parmenter C. A. (2015). Toward a mechanistic understanding of environmentally forced zoonotic disease emergence: sin nombre hantavirus. *BioScience*.

[13] He J., Wang Y., Mu D. (2019). The impacts of climatic factors and vegetation on hemorrhagic fever with renal syndrome transmission in China: a study of 109 counties. *International Journal of Environmental Research and Public Health*.

[14] Ferro I., Bellomo C. M., López W. (2020). Hantavirus pulmonary syndrome outbreaks associated with climate variability in Northwestern Argentina, 1997–2017. *PLOS Neglected Tropical Diseases*.

[15] Tian H., Yu P., Cazelles B. (2017). Interannual cycles of Hantaan virus outbreaks at the human–animal interface in Central China are controlled by temperature and rainfall. *Proceedings of the National Academy of Sciences*.

[16] Xiao H., Tian H.-Y., Cazelles B. (2013). Atmospheric moisture variability and transmission of hemorrhagic fever with renal syndrome in Changsha City, Mainland China, 1991–2010. *PLOS Neglected Tropical Diseases*.

[17] Liu C., Yuan Y., Tao W., Lyu Y., Gao Q. (2022). Enlightenment to the prevention and control of hemorrhagic fever with renal syndrome in field training soldiers by analyzing spatial and temporal distribution in China from 2011 to 2020. *Disease Surveillance*.

[18] Beck H. E., Zimmermann N. E., McVicar T. R., Vergopolan N., Berg A., Wood E. F. (2020). Publisher correction: present and future Köppen–Geiger climate classification maps at 1-km resolution. *Scientific Data*.

[19] Wu H., Wu C., Lu Q., Ding Z., Xue M., Lin J. (2020). Spatial-temporal characteristics of severe fever with thrombocytopenia syndrome and the relationship with meteorological factors from 2011 to 2018 in Zhejiang Province, China. *PLOS Neglected Tropical Diseases*.

[20] Wood S. N., Pya N., Säfken B. (2016). Smoothing parameter and model selection for general smooth models. *Journal of the American Statistical Association*.

[21] Gasparrini A. (2011). Distributed lag linear and nonlinear models in R: the package dlnm. *Journal of Statistical Software*.

[22] Wei Y., Wang Y., Li X. (2018). Meteorological factors and risk of hemorrhagic fever with renal syndrome in Guangzhou, southern China, 2006–2015. *PLOS Neglected Tropical Diseases*.

[23] Wang N., Yin J.-X. (2022). Epidemic process and influencing factors of hemorrhagic fever with renal syndrome: a review. *Chinese Journal of Schistosomiasis Control*.

[24] Xiao H., Tian H.-Y., Gao L.-D. (2014). Animal reservoir, natural and socioeconomic variations and the transmission of hemorrhagic fever with renal syndrome in Chenzhou, China, 2006–2010. *PLoS Neglected Tropical Diseases*.

[25] Huang C., Liu Q. (2022). Interpretation of IPCC AR6 on climate change and human health. *Climate Change Research*.

[26] Luo Y., Lv H., Yan H. (2022). Meteorological change and hemorrhagic fever with renal syndrome epidemic in China, 2004–2018. *Scientific Reports*.

[27] Xu Q.-Q., Li R.-Z., Luo C. (2018). Relationship between meteorological factors and hemorrhagic fever with renal syndrome in Weifang. *Journal of Environmental Health*.

[28] She K., Li C., Qi C. (2021). Epidemiological characteristics and regional risk prediction of hemorrhagic fever with renal syndrome in Shandong Province, China. *International Journal of Environmental Research and Public Health*.

[29] Jiang F., Wang L., Wang S. (2017). Meteorological factors affect the epidemiology of hemorrhagic fever with renal syndrome via altering the breeding and hantavirus-carrying states of rodents and mites: a 9 years’ longitudinal study. *Emerging Microbes & Infections*.

[30] Wang Q., Yue M., Yao P. (2021). Epidemic trend and molecular evolution of HV family in the main hantavirus epidemic areas from 2004 to 2016, in P.R. China. *Frontiers in Cellular and Infection Microbiology*.

[31] Fei L., Wang Z.-G., Yao Y., Xu X.-M., Gu P.-Q. (2015). Population change of farmland rodent and the influences of climate and cultivation factors in Fengxian district of Shanghai. *Chinese Journal of Applied Ecology*.

[32] Joshi Y. P., Kim E.-H., Cheong H.-K. (2017). The influence of climatic factors on the development of hemorrhagic fever with renal syndrome and leptospirosis during the peak season in Korea: an ecologic study. *Bmc Infectious Diseases*.

[33] Tan J., Huang X., Huang P., Liang L. (2022). Epidemiological characteristics of hemorrhagic fever with renal syndrome and its relationship with meteorological factors in Guangdong from 2015 to 2021. *Chinese General Practice*.

[34] Yan L., Liu W., Huang H.-G. (2007). Landscape elements and Hantaan virus-related hemorrhagic fever with renal syndrome, People’s Republic of China. *Emerging Infectious Diseases*.

[35] Teng J., Ding S., Zhang H., Wang K., Hu X. (2023). Bayesian spatiotemporal modelling analysis of hemorrhagic fever with renal syndrome outbreaks in China using R-INLA. *Zoonoses and Public Health*.

