# Supplementary material for: Assessing the Relationship between Climate Variables and Hemorrhagic Fever with Renal Syndrome Transmission in Eastern China: A Multi-Cities Time Series Study
Source: Transbound Emerg Dis. 2023 Sep 20;2023:5572334. doi: 10.1155/2023/5572334 (PMC12016773; doi:10.1155/2023/5572334)
Supplement: Supplementary Materials — Table S1: descriptive statistics of weekly HFRS cases and meteorological factors. Table S2: model test of the interaction analysis. Figure S1: time series plot of HFRS cases and meteorological factors. Figure S2: exposure-response curves for the effects of meteorological factors on weekly HFRS cases in the multiple-variables model using GAM. The x-axis is the meteorological parameters. The y-axis indicates the contribution of the smoother to the fitted values. Figure S3: sensitivity analysis for DLNM by setting the degree of freedom of mean temperature to 4. Figure S4: sensitivity analysis for DLNM by setting the degree of freedom of relative humidity to 4. Figure S5: sensitivity analysis for DLNM by setting the degree of freedom of the week to 7. Figure S6: sensitivity analysis for DLNM by setting the ing degree of freedom of week to 9. Figure S7: sensitivity analysis for DLNM by including mean windspeed. [file 5572334.f1.docx]

**Table S1**

**Descriptive statistics of weekly HFRS cases and meteorological factors**

| Variables | Mean | SD | Min | P25 | P50 | P75 | Max |
| --- | --- | --- | --- | --- | --- | --- | --- |
| Weekly cases | 12.8 | 12.9 | 1.0 | 6.0 | 9.0 | 14.0 | 92.0 |
| Mean temperature (℃) | 13.8 | 9.9 | -7.6 | 4.1 | 14.6 | 23.1 | 29.4 |
| Mean atmospheric pressure (hpa) | 995.6 | 8.5 | 978.8 | 988.1 | 996.3 | 1003.2 | 1013.4 |
| Sunshine duration (h) | 4.4 | 1.4 | 1.5 | 3.1 | 4.4 | 5.6 | 7.4 |
| Mean wind speed (m/s) | 2.9 | 0.7 | 1.6 | 2.5 | 2.8 | 3.3 | 4.6 |
| Mean relative humidity (%) | 63.6 | 12.3 | 33.4 | 54.9 | 63.8 | 73.0 | 88.9 |
| Mean precipitation (mm) | 1.8 | 2.7 | 0.0 | 0.1 | 0.6 | 2.6 | 21.4 |

**Table S2**

**Model test of the interaction analysis**

| Variables | Edf | Ref.df | F-value | P-value |
| --- | --- | --- | --- | --- |
| Model 1 |  |  |  |  |
| MT | 3.727 | 3.933 | 29.246 | <0.001 |
| RH | 1.255 | 1.464 | 1.932 | 0.219 |
| MT, RH | 2.633 | 3.119 | 7.254 | <0.001 |
| Model 2 |  |  |  |  |
| AP | 3.967 | 3.996 | 25.602 | <0.001 |
| RH | 2.082 | 2.536 | 0.959 | 0.378 |
| AP, RH | 9.046 | 11.179 | 2.998 | <0.001 |
| Model 3 |  |  |  |  |
| SH | 2.8861 | 3.322 | 22.802 | <0.001 |
| RH | 2.452 | 2.910 | 3.916 | 0.008 |
| SH, RH | 9.137 | 11.133 | 3.481 | <0.001 |

Abbreviations: Model1: the interaction analysis between MT and RH; Model 2: the interaction analysis between AP and RH; Model 3: the interaction analysis between SH and RH; MT: mean temperature; RH: relative humidity; AP: atmospheric pressure; SH: sunshine duration; Edf: effective degrees of freedom; Ref.df: reference degrees of freedom; F-value is the value of variables using F test.


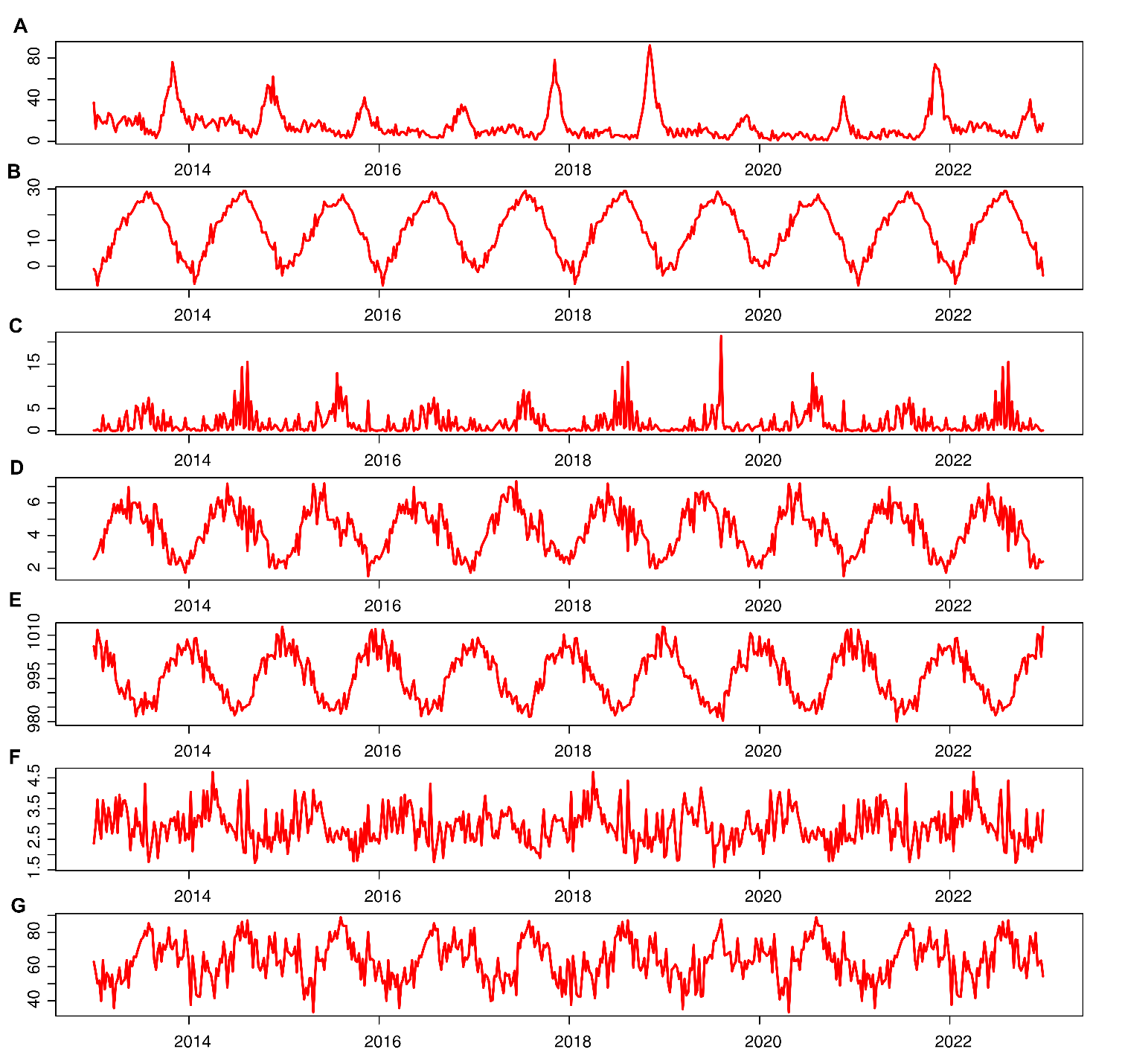


**Fig. S1** **Time series plot of HFRS cases and meteorological factors.** A: weekly total HFRS cases; B: weekly mean temperature; C: weekly mean precipitation; D: weekly mean sunshine duration; E: weekly mean atmospheric pressure; F: weekly mean wind speed; G: weekly mean relative humidity.


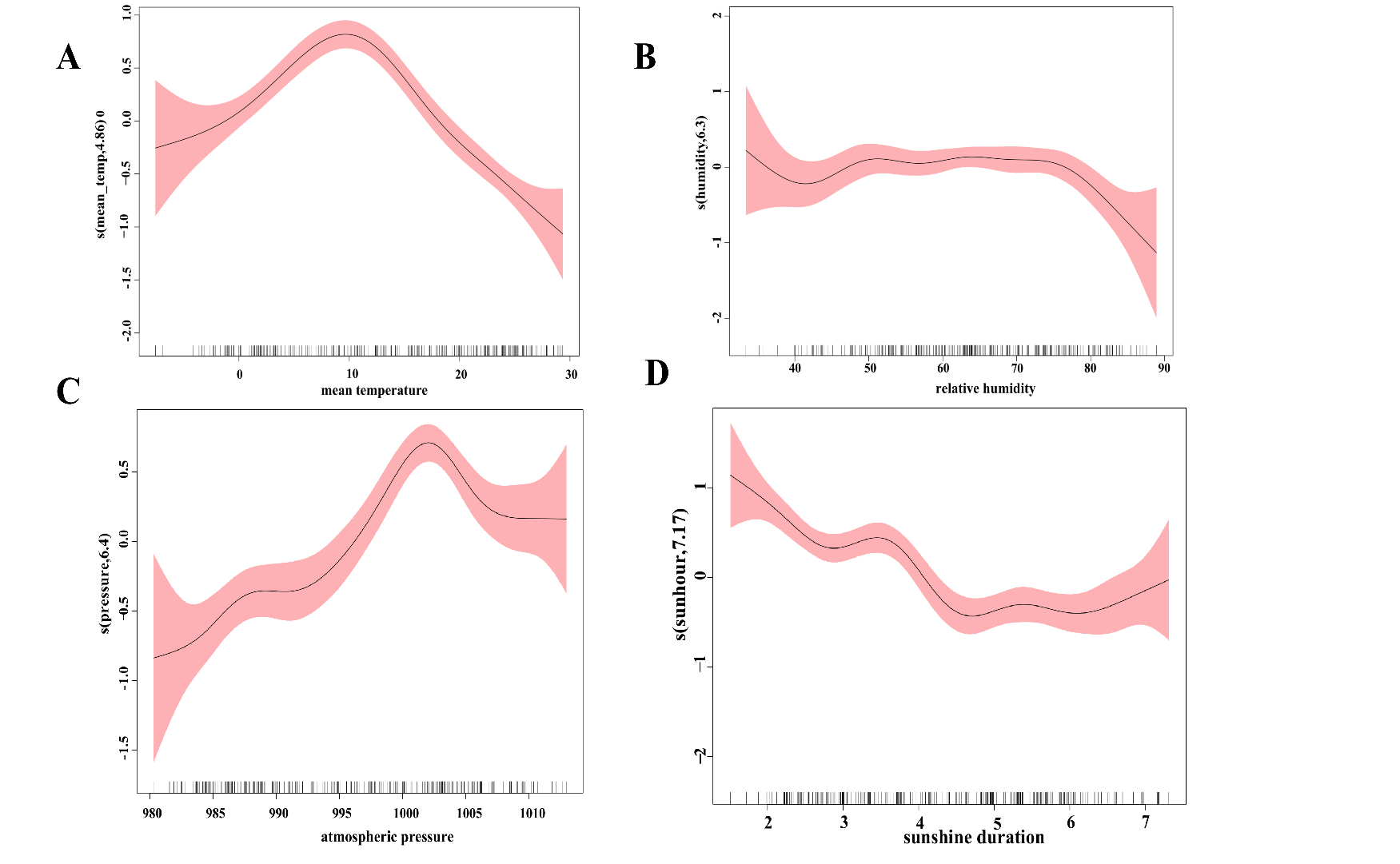


**Fig. S2 Exposure-response curves for the effects of meteorological factors on weekly HFRS cases in the multiple-variables model using GAM**. The x-axis is the meteorological parameters. The y-axis indicates the contribution of the smoother to the fitted values. A: adjusted for relative humidity and windspeed; B: adjusted for the sunshine duration and windspeed; C: adjusted for relative humidity and windspeed; D: adjusted for precipitation and windspeed.


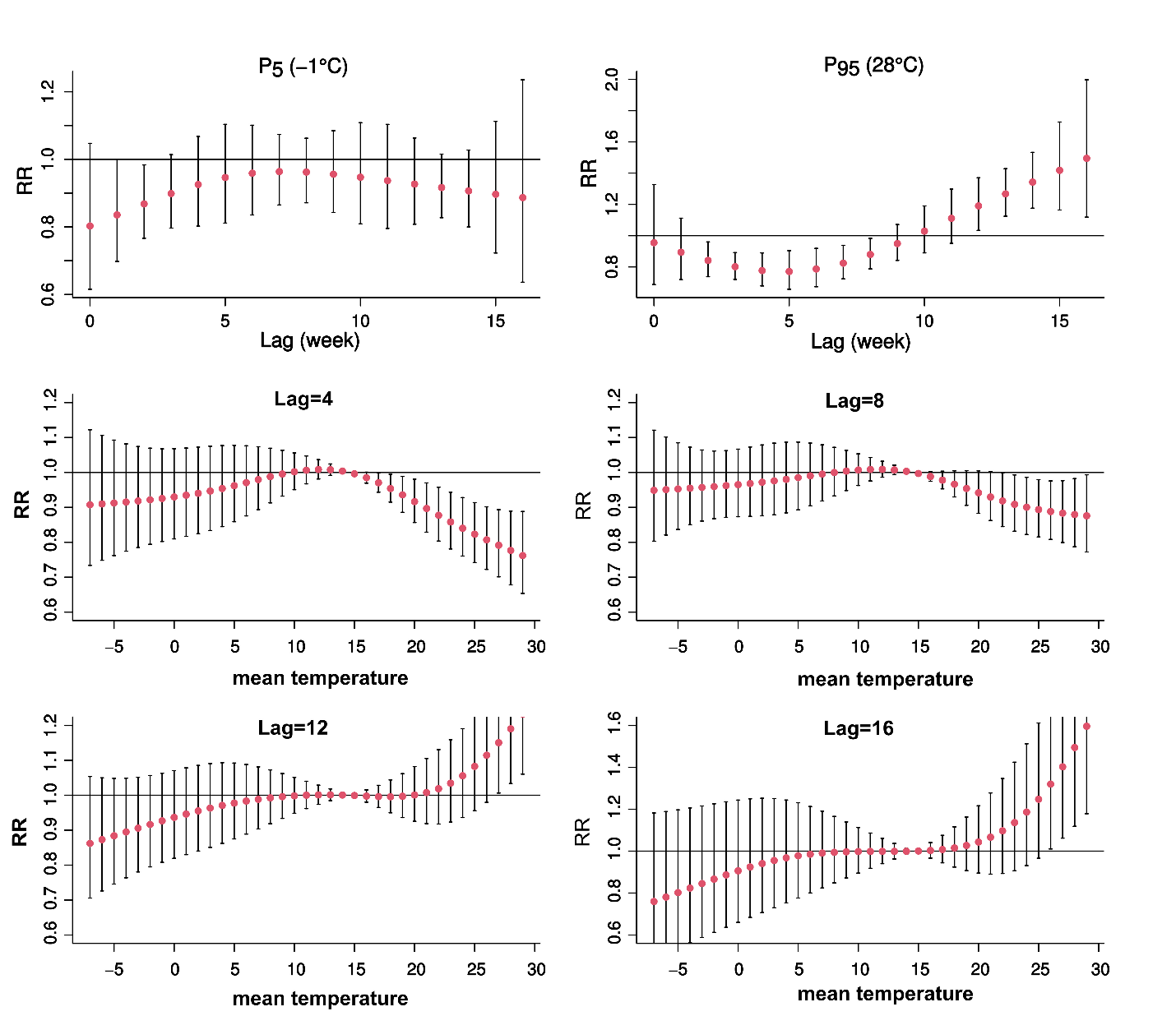


**Fig. S3 Sensitivity analysis for DLNM by setting degree of freedom of mean temperature to 4.** A: exposure-effects of extremely low temperature (-1℃); B: exposure-effects of extremely high temperature (27℃); C: exposure-effects of mean temperature at lag 4th week; D: exposure-effects of mean temperature at lag 8 week; E: exposure-effects of mean temperature at lag 12 week; F: exposure-effects of mean temperature at lag 16th week.


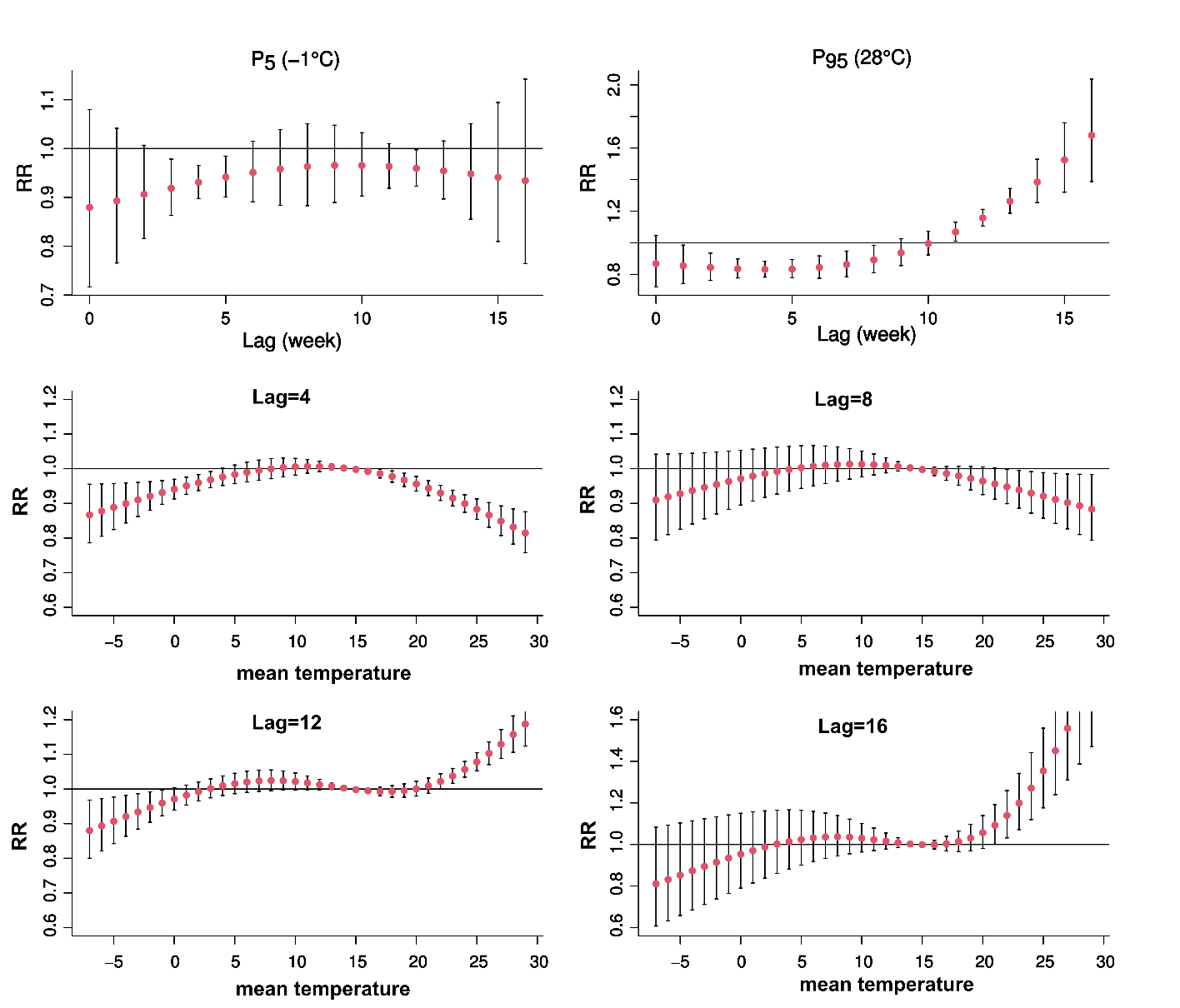


**Fig. S4 Sensitivity analysis for DLNM by setting degree of freedom of relative humidity to 4.** A: exposure-effects of extremely low temperature (-1℃); B: exposure-effects of extremely high temperature (27℃); C: exposure-effects of mean temperature at lag 4th week; D: exposure-effects of mean temperature at lag 8 week; E: exposure-effects of mean temperature at lag 12 week; F: exposure-effects of mean temperature at lag 16th week.


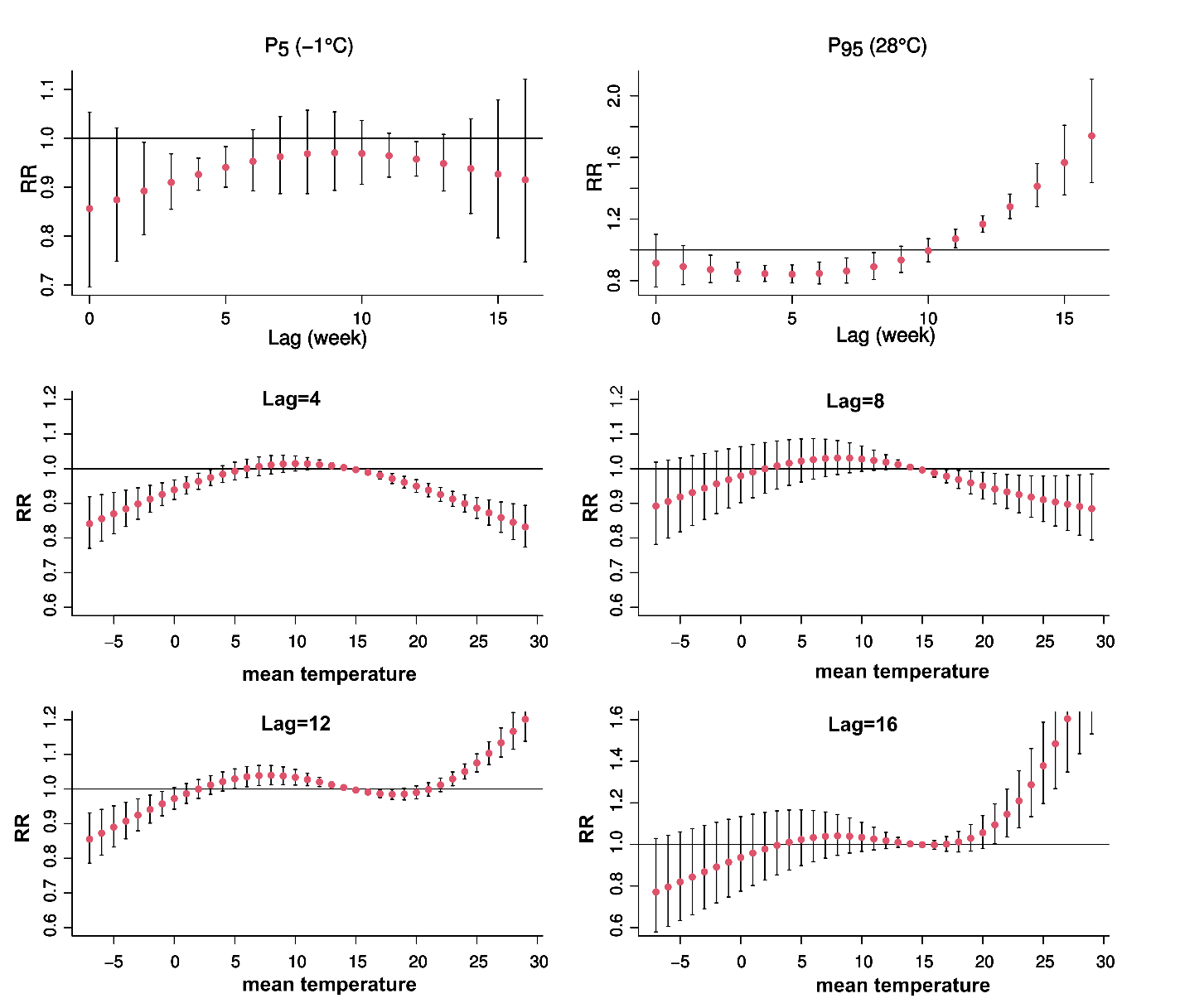


**Fig. S5 Sensitivity analysis for DLNM by setting degree of freedom of week to 7.** A: exposure-effects of extremely low temperature (-1℃); B: exposure-effects of extremely high temperature (27℃); C: exposure-effects of mean temperature at lag 4 week; D: exposure-effects of mean temperature at lag 8th week; E: exposure-effects of mean temperature at lag 12th week; F: exposure-effects of mean temperature at lag 16 week.


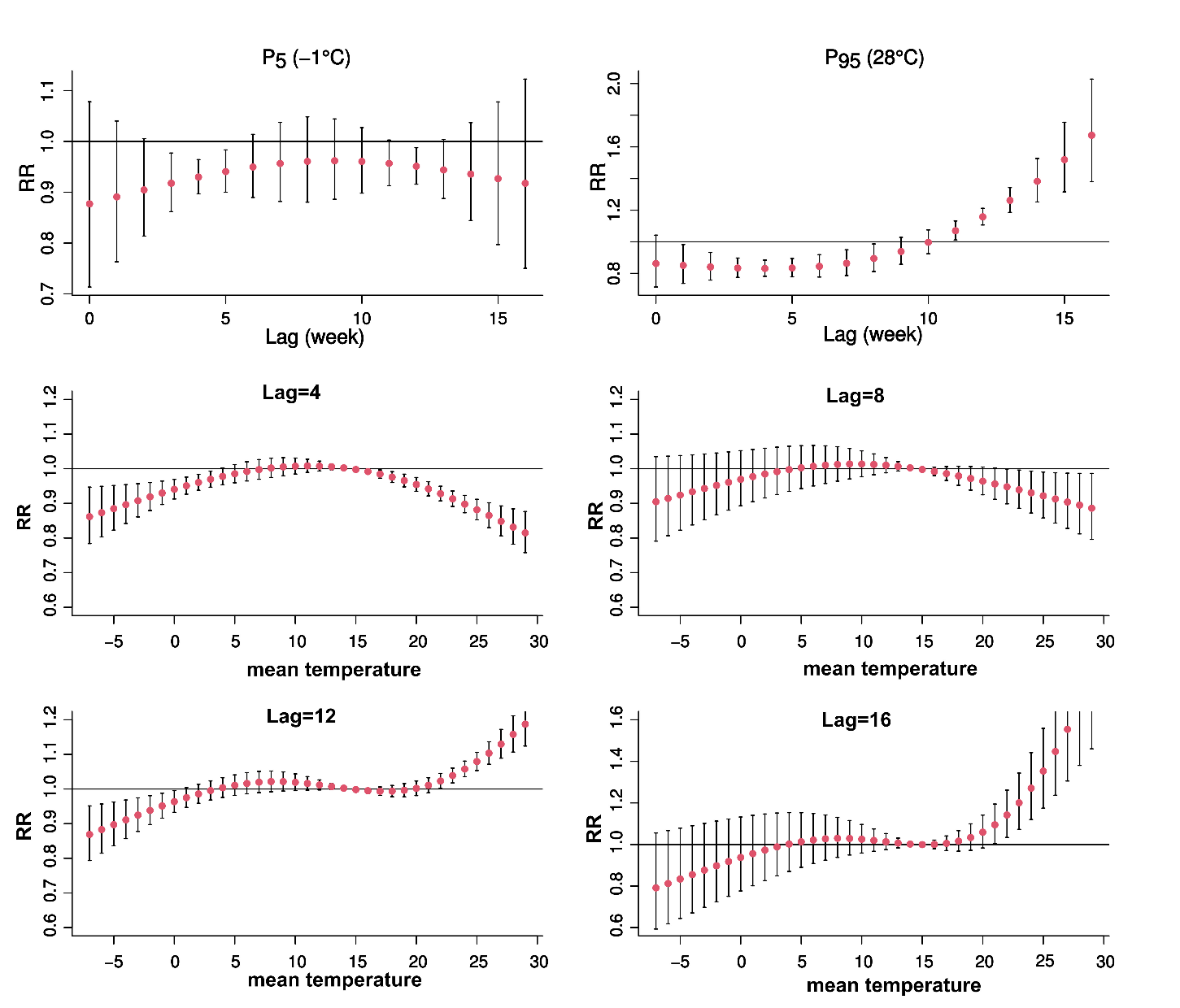


**Fig. S6 Sensitivity analysis for DLNM by setting degree of freedom of week to 9.** A: exposure-effects of extremely low temperature (-1℃); B: exposure-effects of extremely high temperature (27℃); C: exposure-effects of mean temperature at lag 4 week; D: exposure-effects of mean temperature at lag 8th week; E: exposure-effects of mean temperature at lag 12th week; F: exposure-effects of mean temperature at lag 16 week.


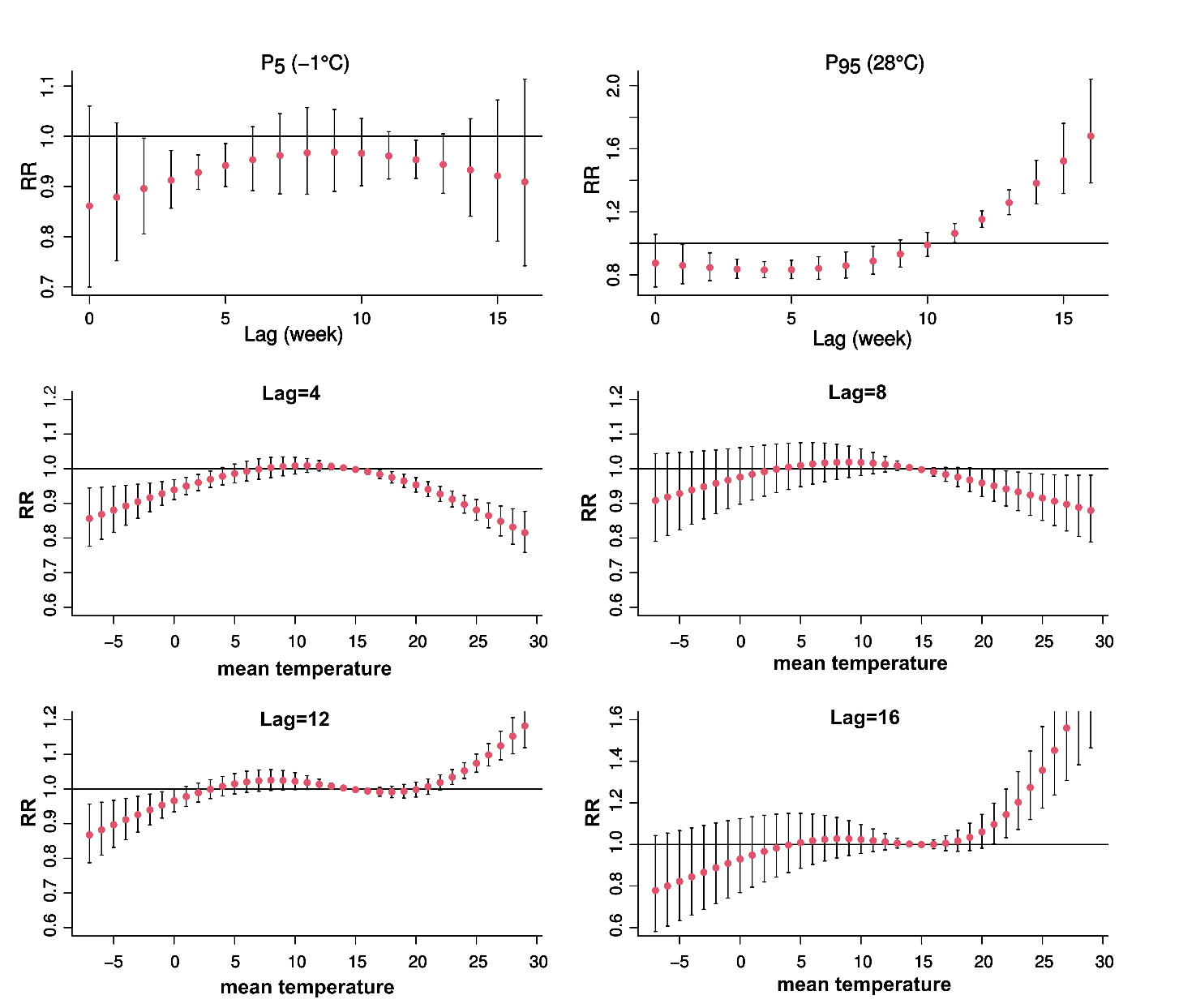


**Fig. S7 Sensitivity analysis for DLNM by including mean windspeed.** A: exposure-effects of extremely low temperature (-1℃); B: exposure-effects of extremely high temperature (27℃); C: exposure-effects of mean temperature at lag 4 week; D: exposure-effects of mean temperature at lag 8th week; E: exposure-effects of mean temperature at lag 12th week; F: exposure-effects of mean temperature at lag 16 week.
